# Association between hemoglobin variants and laboratory outcomes in patients infected with *P. falciparum* from South West Uganda

**DOI:** 10.2144/fsoa-2022-0067

**Published:** 2023-07-17

**Authors:** Hope Onohuean, Fanny Eseohe Onohuean, Ebere Emilia Ayogu

**Affiliations:** 1 Biopharmaceutics unit, Department of Pharmacology & Toxicology, School of Pharmacy, Kampala International University, Western-Campus, Ishaka-Bushenyi, Uganda; 2 Biomolecules, Metagenomics, Endocrine & Tropical Disease Research Group (BMETDREG), Kampala International University, Western Campus, Ishaka-Bushenyi, Uganda; 3 Department of Clinical Pharmacy & Pharmacy Management, University of Nigeria, Nsukka; 4 Department of Clinical Pharmacy & Pharmacy Practice, Kampala International University, Ishaka Uganda

**Keywords:** hemoglobin variants, health facilities, malaria, patients' outcomes, Uganda

## Abstract

**Aims:**

We assess the relationship between various hemoglobin variants and some hematological parameters packed cell volume, white blood cells (PCV, WBC) and parasitemia level of patients with malaria in the southwestern, Uganda.

**Methods:**

Patient were enrolled by rapid diagnostic tests (RDTs), confirmed by microscopy, and laboratory outcomes were determined.

**Results:**

Patients positive for malaria RDTs were 155, microscopic-confirmed *P. falciparum* parasites were 95 (61.29%) having hemoglobin variants HbAA and HbAS; 75 (78.95%) and 13 (13.68%), respectively. The laboratory outcomes showed mean, PCV (32.19 ± 4.83), WBC (5831.66 ± 2888.29) and *P. falciparum* parasitaemia density (32,605.45 ± 14031), while the hemoglobin variants mean values AA (39,008.85 ± 31,261.56), AC (15908 ± 10173.48), AS (16,561.46 ± 15,380.93), SC (30,524 ± 0.000) and SS(1652 ± 0.000) were significantly different from the total population (34,321.5 ± 21,924.26) parasite-density.

**Conclusion:**

Patients with hemoglobin variants HbAA had a significantly higher parasite-carrying capacity and PCV levels.

In endemic countries, malaria continues to be one of the leading causes of illness and mortality, mainly affecting young children [1,2]. Malaria cases increased from 227 million in 2019 to 241 million in 2020, with an estimate 627,000 fatalities globally [3]. About 93% of malaria deaths worldwide occur in 32 sub-Saharan African nations [3]. Increase in malaria infections could further weaken the already over stress health system in many low-income nations with disease burdens such HIV/AIDs, cholera and multi-drug resistance pathogens [1,4,5].

Malaria is a complicated illness that is influenced by numerous unclear host genetic variables [6–9]. The hemoglobin variants like hemoglobin S (HbS), hemoglobin C (HbC), and -thalassemia has been implicated in malaria infestation. Some studies have suggested that some monogenic human diseases confer remarkable levels of protection against severe, life-threatening *falciparum* malaria in African children, with a reducing risk of about 70% in homozygosity HbC and 90% in heterozygosity HbS individuals [9–12]. However, within malaria-endemic areas, hemoglobin S (HbS) has developed into a persistent polymorphism that is linked to a shorter life expectancy in homozygous sickle cell disease sufferers and a longer life expectancy in heterozygous people who are less likely to suffer from malaria [2,13]. However, Africa population have high frequencies of HbS along with other erythrocyte variants that offer defense against the severe illnesses of *P. falciparum* infection While *P. falciparum* have advanced a vast repertoire of genetic mutations to elude the human immune system and resist antimalarial medications [9–12].

It has been found that among patients with malaria, changes in hemoglobin and platelet counts occurred more frequently than changes in WBC counts [14]. Similarly, studies reveals a dramatically decreased counts of neutrophils, monocytes, lymphocytes, and eosinophils as well as red blood cells (RBCs), hemoglobin (Hb), platelets, and white blood cells (WBCs) in patients with malaria [15,16].

Some studies indicated that humans hemoglobin variants co-evolved with *P. falciparum*, such mutations may give inherent resistance to *falciparum* malaria and cause hemoglobinopathies imprinted on the human genome [17–19]. One method for examining human heterogeneities in response to vector infection is the comparison of malaria indicators among groups with various genetic origins that have been consistently exposed to the same parasitic strains [2]. Genetic factor such as hemoglobin genotype may be an important key to understanding and determining susceptibility or resistance to some infectious diseases. Understanding the relationship between different hemoglobin variants and the laboratory outcome (packed cell volume, white blood cell and parasite densities) of malaria in malaria endemic region could advance the knowledge in the treatment of malaria infection and vaccine development. Among the major challenges in malaria control in Uganda include an inadequate understanding of malaria epidemiology, its association with hemoglobin variants and increasing resistance to treatment. In Uganda, there is sparse research on the relationship between different hemoglobin variants and PCV, WBC and *P. falciparum* parasitaemia density among patients with malaria. Such research is relevant in understanding the development mechanism of resistance to approved and effective antimalarial drugs. Therefore, in this study, we evaluate the association between different hemoglobin genotype and the laboratory outcomes of patients with malaria in some selected local health facilities in South West of Uganda.

## Materials & methods

### Study site & population distribution

This study was conducted among patient present with malaria infections in three medical centers (Kitagata Medical Center, Adventist Teaching Hospital and Bushenyi Health Center IV) South Western Uganda. These health centers where randomly selected based on their importance location and the most attend health that met the medical care/need of about 75% of the population in the region.

### Inclusion & exclusion criteria

Patients who were between the ages of 1 month and 65 years, presenting with malaria symptoms such as fever, chill, vomiting, body aches and headaches that agreed to participate via informed consent were recruited. Only patient that tested positive to malaria rapid diagnostic test (RDTs) kit (RDT, OptiMAL) (DiaMed AG, Cressier s/Morat Switzerland) were qualify for inclusion, while those that show negative for the RDT test were excluded.

### Clinical characteristic data collection

The patients' characteristics, such as age, gender, malaria symptoms, patient positive by RDTs, and patient positive by microscopy, were recorded in data collection form in an Excel sheet.

### Sample collection

Blood samples were collected at the clinic from the patients with malaria symptoms and based on preliminary screening that is positive to RDTs. Each patient's 3 ml of venous blood was withdrawn into an EDTA (ethylene diamine tetra acetic acid) container. The tube was appropriately mixed and maintained at 4° for further analysis.

### Parasitological diagnosis

Frosted end slides were used to prepared a smear. The thick and thin films were slides placed on a slide preparation template. The thin film was fixed in absolute methanol for 1 min and allowed to air dried before staining. The dried smears were stained with 3% Giemsa (Sigma-Aldrich, USA) for 40–60 min. The area of the already stained slide that needed to be studied received about two drops of immersion oil. The slide was positioned on the mechanical stage and examined with a 100 × objective light microscope. First, the thick blood film was inspected to count and identify the parasites. Leukocytes and plasmodium parasites were also counted using separate tally counters [20]. Two trained technicians read each film twice, and if there were disparities between the two readers, a third reading was done. As stated in the equation below, the parasite densities were determined by multiplying the patient's absolute WBC count by the parasite count divided by the number of leukocytes counted (i.e., 200 or 500), where a standard number of WBC (8000) was employed.

$$\frac{Number\;of\;parasites\times 8000}{Number\;of\;leukocytes}=Parasites\;per\;microlitre\;of\;blood$$

### Estimation of PCV & WBC

#### Packed cell volume

Special simple capillary glass tubes with an internal diameter of 1 mm and a length of 70 mm were employed. One end of the capillary tubes was sealed with plasticine, and the capillary tubes were two-thirds filled by capillary action with well-mixed venous blood. Using a Haematocrit 210 centrifuge, the sealed capillary tube was spun for five minutes at 1500 r.p.m. (Andreas Hettich GmbH & Co., Germany). The packed cell volume (PCV) was calculated using a hand-held Microhaematocrit Reader (Beckman CoulterTM CSD2, USA) and expressed as a percentage (%) [16,20].

#### White blood cell count

For WBC count, 380 μl of Turks solution was added to 20 μl of blood sample and carefully mixed. A cover slip was used to charge the white blood cell (WBC) cell/μl Neuber counting chamber, and the well was used to pour the diluent into the chamber's cells. The 16 cells' WBC were counted and recorded [21,22].

### Hemoglobin variants

The Helena BioSciences method was used to prepared the cellulose acetate membrane. Each of the outer parts of the electrophoresis chamber received 100 ml of tris-borate-EDTA buffer. To ensure that each support bridge made contact with the buffer and that there were no air bubbles underneath the wicks, two wicks were moistened in the buffer and draped over each one. A cover was placed over the chambers to stop evaporation. In the Zip-Zone well plate, 5 μl of each haemolysate sample test and control were added. The 8unit applicator was used to apply the samples to a cellulose acetate membrane (plate) (Cleaver Scientific Ltd, UK) that had been placed in the Zip-Zone alignment plate. The electrophoresis chamber was immediately plugged in, the cellulose acetate side facing down, and the cellulose acetate membrane was inserted within. The plate was then electrophoresed for 25 minutes. A method called acid citrate agar electrophoresis was employed to separate some of the co-migrating hemoglobins [16,20].

### Statistical analysis

Means percentage was use to analyzed the frequency of patient's clinical characteristic of the study population. The significant epidemiological distribution and risk estimate of malaria hemoglobin variants at 95% confidence interval were evaluated. One-way ANOVA (Turkey test) was used to determine the association between hemoglobin variants and patient laboratory outcome. The statistically significant differences were observed at p < 0.05. All analysis were done in RStudio version 3.5.1 software.

## Results

### Demographic & clinical characteristic of patient's study population

A total of 155 patients that tested positive to malaria RDTs were recruited, 37.42% were between the ages of 1–10 years, female 81 (52.26%), malaria symptoms; fever 140 (90.32%), body pain and tiredness 142 (91.61%). Out of the 155 positives for RDT, 95(61.29%) were positive to microscopy test, the demographic characteristics are shown in Table 1.

**Table 1.** Demographic and clinical characteristic of patients.

Characteristic	Frequency (n)	Percentage (%)
Age (years)		
1–10	58	37.42
11–20	33	21.29
21–30	27	17.42
31–40	11	7.09
41–50	14	9.03
51–60	12	7.74
Total	155	100
Sex		
Female	81	52.26
Male	74	47.74
Total	155	100
Identified malaria symptoms		
Fever	140	90.32
Headache	120	77.42
Shaking chills	125	80.65
Muscle aches or body pain	130	83.87
Tiredness	142	91.61
Vomiting	50	32.26
Diarrhoea	10	6.45
Patients positive by RDTs (n)	155	100
Patients positive by microscopy (n)	95	61.29

RDT: Rapid diagnostic test.

### Epidemiological distribution of malaria infection among different hemoglobin variants in the study population

The epidemiological distribution of malaria infection among the Hb variants showed that (HbAA) 115 (74.20%), (HbAC) 8 (5.20%) and (HbAS) 28 (18.10%) and (HbSS, SC) 2 (1.30%) each among the RDT positive patients detailed in Table 2. Whereas the epidemiological distribution of malaria infection among the Hb variants by the confirmed microscopic positive patients were 75 (78.95%), (HbAC) 5 (5.26%) and (HbAS)13 (13.68%) variants were positive for *P. falciparum* malaria infection, with odd ratio 1.00, 95% CI (69.89–86.26) attributed risk of (aR = 57.89) and relative risk of (RR = 3.75). Also, the HbSS and HbSC variant distribution was 1 (1.05), respectively. The hemoglobin HbAA has a relative risk of (RR = 3.75) detailed in Table 3.

**Table 2.** Distribution of malaria hemoglobin variants among the recruited RDT study population.

hemoglobin variants	Frequency (%)	CI prevalence	RR	aR	Odds ratios	p-value
HbAA	115 (74.20)	66.87–80.62	2.88	48.39	1	–
HbAC	8 (5.20)	2.43–9.56	0.05	-89.68	0.019	0.000
HbAS	28 (18.10)	12.60–24.72	0.22	-63.87	0.077	0.000
HbSS	2 (1.30)	0.22–4.20	0.01	-97.42	0.005	0.000
HbSC	2 (1.30)	0.22–4.20	0.01	-97.42	0.005	0.000

aR: Attributed risk; CI: Confidence interval, LL: Lower limit; RR: Relative risk; UL: Upper limit.

**Table 3.** Distribution of hemoglobin variants among the microscopic positive patients.

Hemoglobin variants	Frequency (%)	CI prevalence	RR	aR	Odds ratios	p-value
HbAA	75 (78.95)	69.89–86.26	3.75	57.89	1	–
HbAC	5 (5.26)	1.95–11.28	0.06	-89.47	0.015	0.000
HbAS	13 (13.68)	7.83–21.73	0.16	-72.63	0.042	0.000
HbSS	1 (1.05)	0.05–5.08	0.01	-97.89	0.003	0.000
HbSC	1 (1.05)	0.05–5.08	0.01	-97.89	0.003	0.000

aR: Attributed risk; CI: Confidence interval; LL: Lower limit; RR Relative risk; UL: Upper limit.

### Association between hemoglobin variants & patient laboratory outcome

The malaria patient's laboratory outcomes showed that the mean ± SEM of PCV, WBC, *P. falciparum* parasitaemia density were 34.19 ± 4.83, 5831.66 ± 2888.29 and 32,605.45 ± 54,079, respectively.

**Figure 1.**
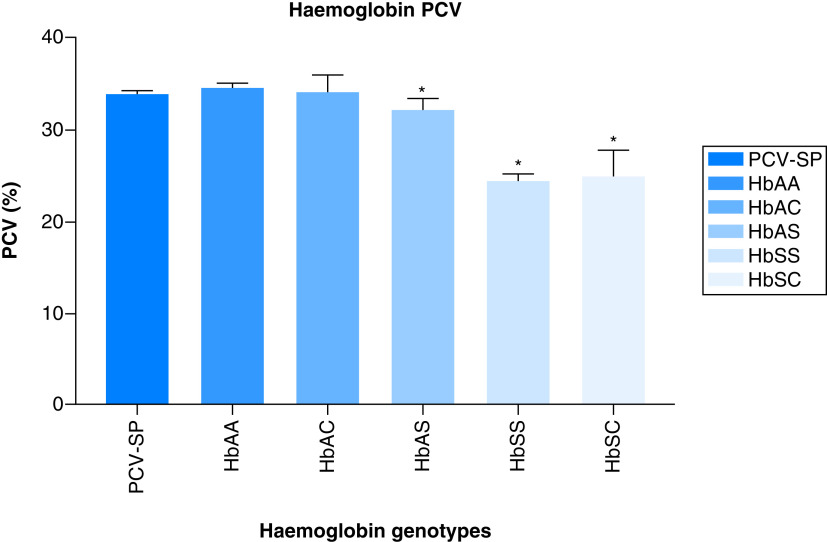
The Association between hemoglobin variants and the patients packed cell volume. PCV: Packed cell volume.

A comparison of how the different variants were associated with the laboratory outcomes showed that for PCV, HbAS, HbSS and HbSC had mean values of (32.59 ± 4.97), (24.5 ±0.71), and 25 ± 2.83), with the (p = 0.0219, 0.0115 and 0.0134), respectively. There was no significant difference (p = 0.0905) in the mean values of patient with Hb AA and AC (34.09 ± 4.17, 34.14 ± 4.09) compared with the PCV mean value (33.89 ± 5.08) of the total population. The mean PCV value of the other hemoglobin variants and their association are shown in Figure 1.

**Figure 2.**
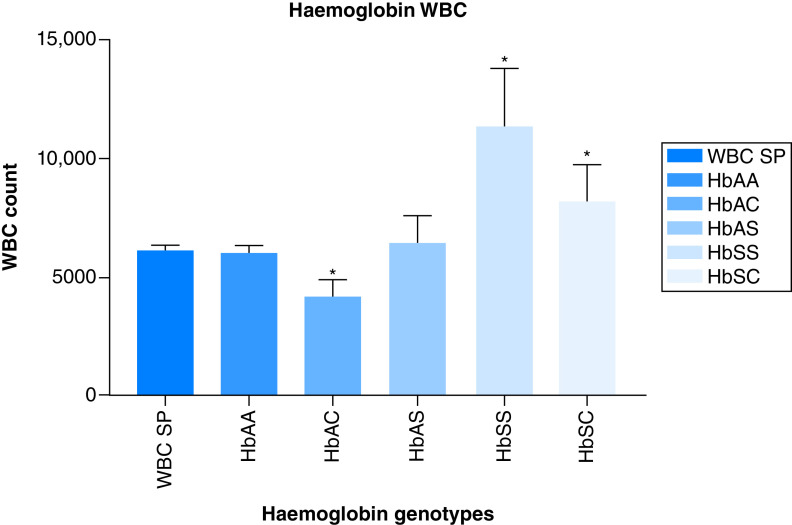
The Association between hemoglobin variants and the patients white blood cells. WBC: White blood cell.

For WBC, The HbAC, HbSS and HbSC had the mean values of (4195.71 ± 1592.09), (11,360 ± 1372.87) and (8310 ± 1654.63), respectively. There was significant difference (p = 0.0121, 0.0013, 0.0105) between the men value of HbSS, SC, AC and the WBC mean value (6138.59 ± 3040.3) of the total population. The other WBC value for other hemoglobin variants with their association are shown in Figure 2.

**Figure 3.**
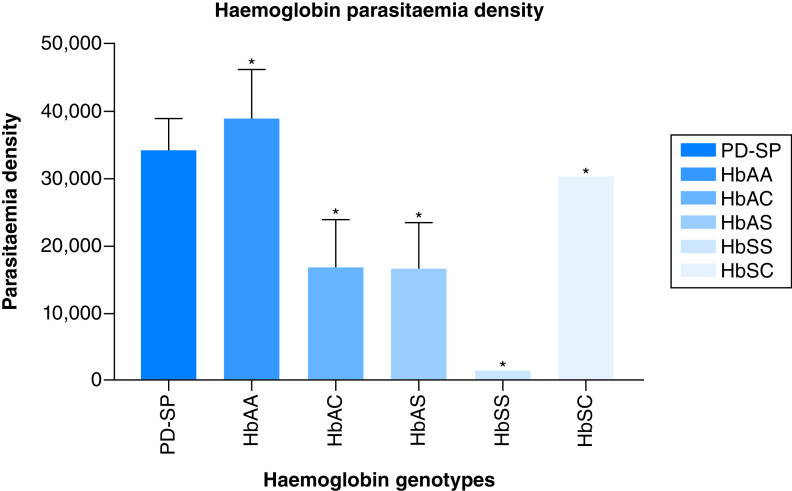
The Association between hemoglobin variants and the *P. falciparum* parasitemia density.

For parasite density, the HbAA, AC, AS, SC and SS has mean mean values of (39,008.85 ± 31,261.56), (15,908 ± 10,173.48), (16,561.46 ± 15,380.93), (30,524 ± 0.000) and (1652 ± 0.000), respectively. There was a significant difference (p = 0.0105, 0.0045, 0.0032, 0.0017, 0.0021, 0.0314)) in the mean value of all the hemoglobin variants compared with that of the total population (34,321.5 ± 21,924.26). The various mean parasite densities of other hemoglobin genotype are shown in Figure 3.

## Discussion

There is an evolving knowledge on the relationship between different hemoglobin variants and malaria parasites [23]. To expand the transforming focus on eliminating malaria, understanding how common genetic variants affect parasite distribution, transmission processes can vary in communities with different frequencies of these alleles would be especially helpful. Here we access the relationship between various hemoglobin variants and the laboratory outcomes of patients with malaria in selected local health facilities in the southwestern region of Uganda. Our finding reveals the most vulnerable age of malaria infection to be between 1–10 years with an utmost complain of feverish and tiredness similar to other studies [24–26]. This further validated that children are most vulnerable to malaria infection [27,28] been that there is little or no well-developed immunity to malaria transmission in the endemic areas of Sub-Sahara Africa.

The relatively low PCV of about 32%, which is less than the standard lowest limits of 36–38%, indicates a potential anaemia condition which is a common finding in malaria, especially children; similar findings have been reported in studies from Keyan and Nigeria [14,15,29]. However, suppression of the immune system or comorbidity of other infections, such as HIV, and TB could be responsible for such findings in the malaria endemic region. We are not surprised at the result of the WBC count as it reveals the haematological parameter changes exhibited in patients infected with malaria [14,30]. It is attributed to physiological variability in individuals, and the acute phase of malaria infection which normalizes to WBC counts trend after disease resolution [14,30,31]. Therefore, WBC counts may be used as a marker for malaria and other disease progression that guide management decisions. The microscopy epidemiological distribution investigation showed that malaria parasite invasion threatened HbAA and HbAS phenotype carriers among the study population. It also implies that the malaria parasite has <1 risk infection in HbSS and HbSC population. However, the severity of the infection in hemoglobin HbAA genotype risk to *P. falciparum* malaria parasites is significant to the study population. Furthermore, the chance of a patient with HbAS becoming malaria undesirable compared with a patient with HbAA is about 4%. The population with HbAS had a lower prevalence of malaria than with HbAA, and this was correlated with a considerably lower level of parasitemia. This finding is in agreement with a number of studies indicate that both hemoglobin genotype C and S offer protection against severe malaria invasion [9,32,33]. Although it's plausible that these variants mechanisms of defense target pathogenic occurrences that really are specific to severe clinical cases, epidemiologic studies proof that these variants also affect non-severe clinical malaria risk implies that heterozygosity for hemoglobin C or S influences pathogenic mechanisms that are shared by malaria infections. Additionally, experimental research suggests that these alterations have a variety of effects on malaria parasites [32,34]. However, these various mechanisms play important roles in natural infections, we still don't fully comprehend how within-host parasite dynamics differ between individuals with AA and AS or AC heterozygotes, particularly the length of parasitic infestation, circulation and the transmission stages.

Our results also indicate variation in the association between hemoglobin variants and patient PCV, WBC, *P. falciparum* parasitaemia density. This is expected as malaria is known to be characterized by hematological abnormalities, which are said to be most prominent in infections with *P. falciparum*. A number of investigations have revealed either a lower prevalence or a lower density of parasitaemia compared with with HbAA [35]. The lower ability of parasites to grow and proliferate in HbAS and HbSS cells could be caused by increased red cell membrane resistance to the invading parasites and a hypoxic environment within the red cell that inhibits their development. The potential mechanisms may be due to the ability of parasite infected HbAS erythrocytes to get sickled six-times more frequently than non-parasitized HbAS cells, either resulting to intracellular parasite death and/or their improved elimination by the enhanced development of malaria-specific immunity (both innateand acquired immunity) [20,36,37]. Also, our study supports earlier findings that a high malaria parasitaemia level in HbAA caused a decrease in reticulocyte count due to dyserythropoietic effect of the parasitaemia's resulted from the malarial pigment or hemozoin [38,39]. An oxidized products, or an imbalance in cytokine response that has an adverse effect on erythropoiesis [40].

This study's limitations included the absence of prior medical histories for conditions such Hb disorders, anemia, bacterial or viral infections, which could potentially skew analysis and alter the interpretation of the findings. Also, the use of estimation methods as set by WHO to that of the hematology analyzers that was not available at the time of this study.

## Conclusion

This study has demonstrated that individuals who had the HbAC, HbAS, HbSS, or HbSS variants, has a variable susceptibility to malaria with the high prevalence of the HbAA phenotype to malaria parasite infection. However, the prevalence of *P. falciparum* parasitaemia in HbAS compared with HbAA patients may corroborate the protective effects that hemoglobinopathies give. The low level of *P. falciparum* parasitaemia in older age groups supports the idea that immunity develops naturally over time in endemic areas. In order to build strategic policies for the creation of efficient malaria control programs aimed at eradicating and/or lowering this life-threatening disease to the bare minimum, this study will help to better understand the endemicity of malaria in the study area.

**Summary points**

- Understanding malaria epidemiology and its association with hemoglobin variants may improve treatment policy.
- Patients with malaria confirmed by microscopy assay were recruited to determine the association between various hemoglobin variants and PCV, WBC, parasitaemia density.
- Findings showed that patients with hemoglobin variants HbAA have a significantly higher parasite-carrying capacity.
- The low level of *P*. *falciparum* parasitaemia in HbAS may corroborate with the protective effects offered by hemoglobinopathies.
